# Clinical features, fungal load, coinfections, histological skin changes, and itraconazole treatment response of cats with sporotrichosis caused by *Sporothrix brasiliensis*

**DOI:** 10.1038/s41598-018-27447-5

**Published:** 2018-06-13

**Authors:** Elaine Waite de Souza, Cintia de Moraes Borba, Sandro Antonio Pereira, Isabella Dib Ferreira Gremião, Ingeborg Maria Langohr, Manoel Marques Evangelista Oliveira, Raquel de Vasconcellos Carvalhaes de Oliveira, Camila Rocha da Cunha, Rosely Maria Zancopé-Oliveira, Luisa Helena Monteiro de Miranda, Rodrigo Caldas Menezes

**Affiliations:** 1 Evandro Chagas National Institute of Infectious Diseases, Oswaldo Cruz Foundation, Rio de Janeiro, Brazil; 2 Oswaldo Cruz Institute, Oswaldo Cruz Foundation, Rio de Janeiro, Brazil; 3 School of Veterinary Medicine, Louisiana State University, Baton Rouge, LA United States of America

## Abstract

Zoonotic sporotrichosis caused by the fungus *Sporothrix brasiliensis* is usually severe in cats. This study investigated the associations between clinical features, fungal load, coinfections, histological skin changes, and response to itraconazole in cats with sporotrichosis caused by *S*. *brasiliensis*. Fifty-two cats with skin lesions and a definitive diagnosis of sporotrichosis were treated with itraconazole for a maximum period of 36 weeks. The animals were submitted to clinical examination and two subsequent collections of samples from the same skin lesion for fungal diagnosis and histopathology, as well as serology for feline immunodeficiency (FIV) and leukaemia (FeLV) viruses. Thirty-seven (71%) cats were clinically cured. Nasal mucosa lesions and respiratory signs were associated with treatment failure. Cats coinfected with FIV/FeLV (n = 12) had a lower neutrophil count in the lesion. A high fungal load in skin lesions was linked to young age and treatment failure, as well as to a longer time of wound healing, poorly formed granulomas and fewer neutrophils, macrophages and lymphocytes in these lesions. These results indicate that itraconazole is effective, but nasal mucosal involvement, respiratory signs and high fungal loads in skin lesions are predictors of treatment failure that will assist in the development of better treatment protocols for cats.

## Introduction

Sporotrichosis is a widely distributed worldwide mycosis that usually affects the skin and subcutaneous tissue of humans and several animal species, but that can spread to other organs^1–3^. The main fungal species causing this disease are *Sporothrix schenckii sensu stricto*, *S. brasiliensis*, *S. globosa*, and *S. luriei*^4,5^. Infection by the fungus *Sporothrix* spp. may occur through animal-animal, zoonotic or sapronotic transmission by traumatic inoculation of the fungus into skin^5^.

Zoonotic sporotrichosis is hyperendemic in the metropolitan region of the state of Rio de Janeiro, Brazil, and is transmitted mainly by scratches and bites from infected domestic cats^6–8^. In this region, the species identified in feline cases and in most human cases is *S. brasiliensis*^9–11^. This fungal species has also been described in cases of zoonotic sporotrichosis transmitted by cats in other Brazilian states^10–14^.

In cats, the most common clinical form of sporotrichosis are multiple skin nodules and ulcers, frequently associated with nasal mucosa lesions and respiratory signs^15,16^. Systemic involvement, poorly formed granulomatous inflammation, and large numbers of intralesional yeasts are commonly seen^1,15,17^. Additionally, low response rates to treatment with antifungal drugs including itraconazole, the drug of choice for this disease, are often reported^16^. Taken together, these characteristics indicate a high susceptibility of cats to sporotrichosis, in contrast to humans and dogs with sporotrichosis in which systemic involvement is rare and the response to treatment is usually good^2,18^.

Although difficult-to-treat forms of sporotrichosis are common in cats, little is known about predictors of the treatment response in animals with sporotrichosis caused by *S. brasiliensis*. This fungal species is considered the most virulent within the genus *Sporothrix*^19,20^ and may be associated with the high frequency of severe lesions in the nasal mucosa and of upper respiratory signs in cats from Rio de Janeiro^3,16^. Within this context, respiratory signs have been correlated with treatment failure and death in cats with sporotrichosis^16^. In contrast, treatment failure does not seem to be associated with infection with feline immunodeficiency virus (FIV) or feline leukaemia virus (FeLV)^16^. Good body condition, localised lesions, and the presence of well-formed granulomas in the skin lesions are linked to lower fungal load and are therefore suggestive of a better outcome of the disease in cats with sporotrichosis^17^. However, these previous studies have not identified the species *S. brasiliensis* because phenotypical and molecular characterization of the fungus was not performed^3,16,17^. In addition, these studies have not evaluated the association between clinical features, fungal load, coinfection with FIV/FeLV and histological skin changes before and during the treatment as predictors of itraconazole treatment response in cats^3,16,17^. The identification of these predictors will assist in the development of better treatment protocols for feline sporotrichosis and in the prevention of zoonotic transmission of this disease. Therefore, the aim of this study was to investigate for the first time the associations between clinical features, fungal load, coinfection with FIV/FeLV, histological skin changes, and treatment response to itraconazole as predictors of outcome in cats with sporotrichosis caused by *S. brasiliensis*.

## Results

### Clinical examination and treatment outcome

The 52 animals included in the study were domestic short-haired cats, with a median age of 3 years (range 1 to 10 years). The median weight was 4 kg (range 2 to 7 kg). Thirty-eight (73%) animals were males.

**Figure 1:**
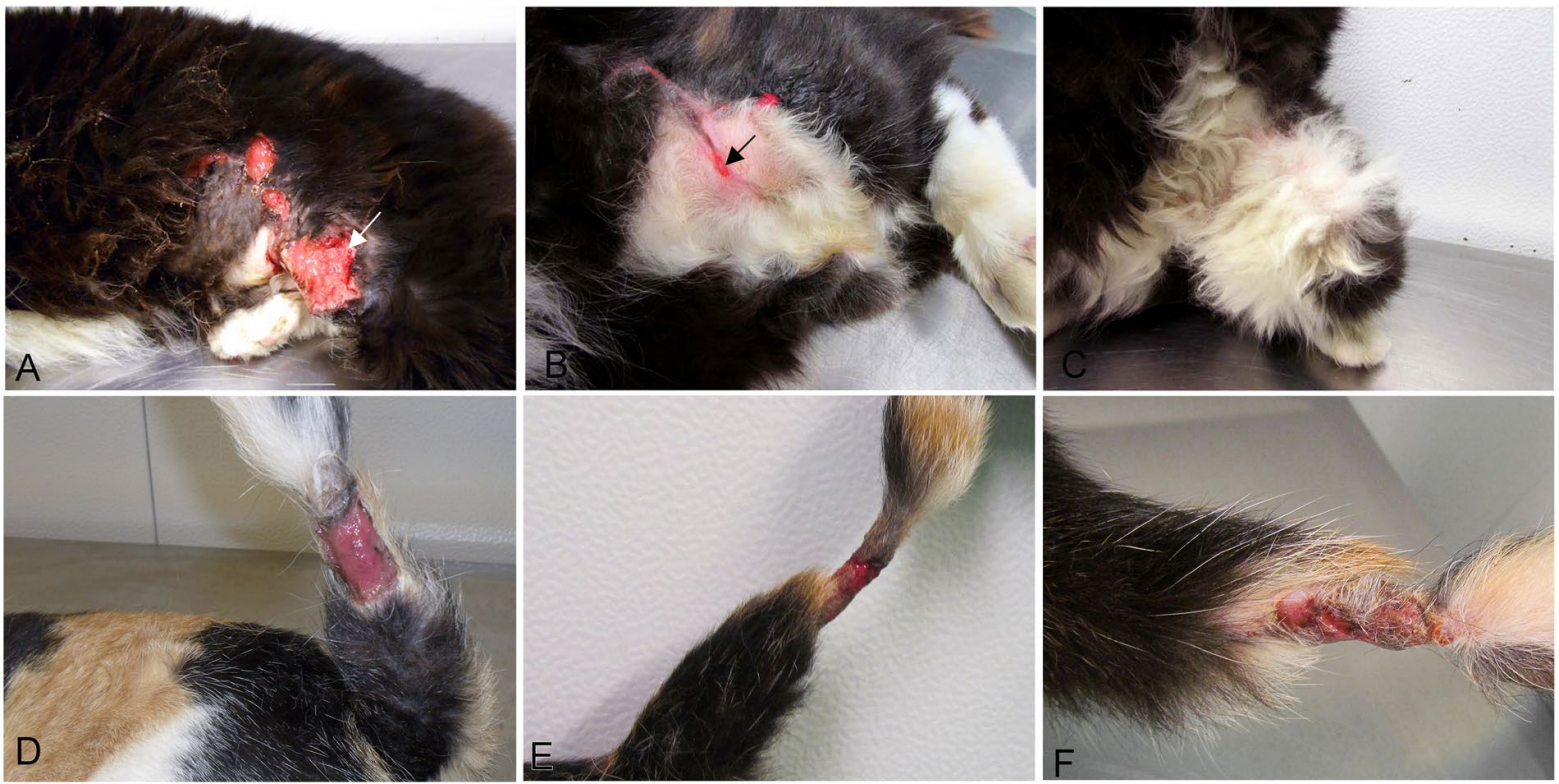
(**A**–**C**) Female cat, 5 years, with sporotrichosis and negative serology for FIV and FeLV. (**A**) Multiple ulcers on the flank and thigh in the first appointment before treatment with itraconazole. One of the lesions of the flank (arrow) was submitted to the first biopsy. (**B**) Time of the second biopsy of the same lesion (arrow) after 7 weeks of treatment. Remarkable regression of size and partial healing of the ulcer on the right flank submitted to the first biopsy, as well as of the other initial lesions. (**C**) Clinical cure after 16 weeks of treatment with itraconazole. (**D–F**) Female cat, 3 years, with sporotrichosis and negative serology for FIV and FeLV. (**D**) Ulcer on the tail before treatment with itraconazole submitted to the first biopsy. (**E**) Time of the second biopsy of the same lesion after 9 weeks of treatment. Note the stagnation of the ulcer on the tail. (**F**) Therapeutic failure after 36 weeks of itraconazole treatment. Persistence of the ulcer on the tail.

Clinical cure was observed in 37 (71%) cats (Fig. 1A–C) and treatment failure occurred in 15 (29%) animals (Fig. 1D–F). The median healing time of the skin lesion examined and the duration of treatment in clinically cured cats were 8 and 16 weeks, respectively.

There was a significant reduction in the number and distribution of lesions between the time of the first appointment before treatment with itraconazole (T1) and the time of the second biopsy of the same skin lesion between the 5th and 11th week after the beginning of itraconazole treatment (T2) (Table 1). The frequency of nasal mucosa lesions and respiratory signs was the same at T1 and T2 (Table 1) and was significantly associated with treatment failure (p = 0.027, Fisher’s exact test). The frequency of treatment failure was higher among cats with respiratory signs and nasal mucosa lesion (13 of 32 animals) than among those without respiratory signs (2 of 20 animals). Fisher’s exact test showed no significant association of body condition (p = 0.096 at T1 and p = 0.26 at T2), lesion distribution (p = 0.33 at T1 and p = 0.024 at T2), or number of lesions (p = 0.132 at T1 and p = 0.067 at T2) with treatment outcome (clinical cure vs treatment failure) at either time point (T1 and T2). The diameter of the biopsied skin lesion was only measured at T1; 28 (54%) cats had lesions smaller than 2 cm and 24 (46%) had lesions larger than 2 cm. There was no association between the diameter of the biopsied skin lesion and treatment outcome (p = 0.363, Fisher’s exact test).

**Table 1:** Clinical features of the 52 cats with sporotrichosis in the first appointment before treatment with itraconazole (T1) and in the appointment between the 5th and 11th week after the beginning of itraconazole treatment (T2).

Clinical features	Classification	Number of cats (n = 52)		p^b^
		T1	T2	
Body condition	Good	37	41	0.344
	Fair/poor	15	11	
Distribution of skin lesions	L1/L2	17	34	<0.001*
	L3	35	18	
Number of skin lesions	≤3	23	48	<0.001*
	>3	29	4	
Nasal mucosa lesions	Yes	32	32	1.000
	No	20	20	
Respiratory signs^a^	Yes	32	32	1.000
	No	20	20	

L1, lesions at one site; L2, lesions at 2 non-contiguous sites; L3, lesions at 3 or more non-contiguous sites; n, total number of cats examined. ^a^Sneezing, nasal discharge or dyspnoea; ^b^McNemar test. *Significant difference (p < 0.05).

### Identification of the *Sporothrix* species

All 52 fungal isolates were identified as *S. brasiliensis*. Three isolates could not be identified to species level by the phenotyping method based on sugar assimilation since they did not ferment sucrose. These three isolates were thus identified as *S. brasiliensis* by PCR fingerprinting with the T3B primer.

Among the 52 *S. brasiliensis* isolates identified, a predominance of hyaline conidia or the same amount of hyaline and pigmented conidia was observed in 33 (63.5%) isolates, and a predominance of pigmented conidia was found in 19 (36.5%). Pigmented conidia indicate the presence of melanin. There was no significant association between the predominance of pigmented conidia and treatment outcome (13 cases of clinical cure and 6 cases of treatment failure; p = 0.76, Fisher’s exact test).

### Coinfection of *Sporothrix* with FIV and FeLV

Ten (21%) of the 48 cats tested were seropositive for FeLV, two (4%) were seropositive for FIV, and none of the animals tested seropositive for both viruses. Eight FeLV-seropositive cats and one FIV-seropositive cat were clinically cured. Treatment failure occurred in two FeLV-seropositive cats and in one FIV-seropositive cat. No significant association was observed between coinfection and treatment outcome (p = 1.0, Fisher’s exact test) or healing time of the skin lesion examined (p = 0.698, Mann-Whitney test).

### Histological changes

Histopathological analysis of the skin lesion samples biopsied at T1 revealed a pyogranulomatous infiltrate in all 52 cats with sporotrichosis. With respect to granuloma organisation, a poorly formed granuloma was observed in 47 (90%) cats and a well-formed granuloma in 5 (10%). Among cats with poorly formed granulomas, the treatment outcome was clinical cure in 32 and therapeutic failure in 15. All 5 cats with well-formed granulomas were clinically cured. Regarding the type of granuloma, the poorly formed granuloma containing macrophages packed with yeasts and a sparse lymphoplasmacytic infiltrate was classified as fungus-rich granuloma. Both the poorly formed granuloma without the characteristics of fungus-rich granulomas described above and the well-formed granuloma were classified as typical granuloma. Typical granulomas were observed in 37 (71%) cats and fungus-rich granulomas in 15 (29%). The type of granuloma was significantly associated with treatment outcome (p = 0.020, Fisher’s exact test). There was a higher frequency of treatment failure in cats with fungus-rich granulomas (8 of 15 animals) when compared to cats with typical granulomas (7 of 37 animals). Round or cigar-shaped yeasts and hyphae or pseudohyphae compatible with *Sporothrix* spp. were observed in 47 (90%) animals. Dermal fibrosis was found in 2 (4%) cats and clinical cure was the outcome in both of them.

Table 2 shows the median and range of the number of inflammatory cells and fungal load per mm^2^ in the skin lesion sample biopsied at T1 of the 52 cats included in the study according to outcome of itraconazole treatment and positive serology for FIV and FeLV. The fungal load was significantly higher among cats with treatment failure and the number of neutrophils was significantly higher among those seronegative for FIV/FeLV (Table 2).

**Table 2:** Median (range) number of inflammatory cells and fungal load per mm^2^ in the skin lesion sample biopsied at T1 of the 52 cats with sporotrichosis according to outcome of itraconazole treatment and positive serology for FIV or FeLV.

Cells/yeasts	Median (range) number of cells/mm^2^ in the biopsied skin lesion						
	Total (n = 52)	Outcome (n = 52)		p^a^	FIV/FeLV (n = 48)		p^a^
		TF (n = 15)	CC (n = 37)		Positive (n = 12)	Negative (n = 36)	
Neutrophils	171.1 (29.6–381.0)	139.5 (53.2–234.4)	152.7 (47.6–239.2)	0.785	119.4 (47.6–173.4)	144.2 (87.4–239.2)	0.021*
Macrophages	131.0 (9.0–1576.0)	121.6 (80.6–157.2)	122.2 (65.4–175.2)	0.317	105.6 (80.6–170.6)	127.4 (65.4–175.2)	0.938
Lymphocytes	29.6 (13–106.4)	29.8 (14.0–106.4)	29.6 (18.4–41.8)	0.793	30.6 (18.0–40.0)	25.8 (14.0–106.4)	0.482
Plasma cells	13.8 (0–89.2)	11.6 (0.6–46.8)	15.6 (0–89.2)	0.739	15.6 (3.8–33.0)	11.0 (0–89.2)	0.543
Mast cells	9.4 (0–28.2)	7.7 (0.6–12.6)	9.7 (0.8–20.8)	0.443	8.5 (3.2–20.8)	9.2 (0.6–15.2)	0.858
MGC	0 (0–3.4)	0 (0–0.2)	0 (0–3.4)	0.844	0 (0–0.2)	0 (0–3.4)	0.858
Eosinophils	0 (0–5.6)	0 (0–1.8)	0 (0–1.6)	0.87	0 (0–1.2)	0 (0–1.8)	0.674
Yeasts	38.1 (0–1,274.8)	745.5 (2.2–1,274.8)	204.9 (0.8–928.0)	0.002*	717.1 (10.0–1,274.8)	309.2 (0.8–864.0)	0.095

MGC, multinucleated giant cells; n, number of cats; TF, treatment failure; CC, clinical cure; ^a^Mann-Whitney test. *Significant difference (p < 0.05).

In skin lesion samples biopsied at T1, the fungal load was significantly higher (p < 0.001, Mann-Whitney test) in fungus-rich granulomas (median 829.6 yeasts/mm^2^, range 621.0–1274.8) than in typical granulomas (median 10.0 yeasts/mm^2^, range 0.8–833.0). Additionally, the fungal load was significantly lower (p = 0.002, Mann-Whitney test) in well-formed granulomas (median 1.4 yeasts/mm^2^, range 0.8–2.0) than in poorly formed granulomas (median 506.2 yeasts/mm^2^, range 1.4–1274.8). Fisher’s exact test revealed no significant association of positive serology for FIV/FeLV with granuloma type (p = 0.726) or organisation (p = 0.312). Ten of the 14 cats with fungus-rich granulomas tested negative for FIV and FeLV. On the other hand, all 5 cats with well-formed granulomas tested negative for FIV and FeLV. Using Spearman’s rank correlation coefficient, a negative correlation was observed between fungal load and the number of lymphocytes (r = −0.280, p = 0.044), neutrophils (r = −0.369, p = 0.007), and macrophages (r = −0.344, p = 0.012). Additionally, the healing time of the lesion examined was positively correlated with fungal load (r = 0.428, p = 0.002) and negatively with the number of neutrophils (r = −0.522, p < 0.001).

The age of the cats was positively correlated with the number of lymphocytes (r = 0.334, p = 0.016) and negatively with fungal load (r = −0.281, p = 0.043) in skin lesion samples biopsied at T1 using Spearman’s rank correlation coefficient. The median age of cats with fungus-rich granulomas was lower (median 2.0 years, range 1.0–6.0) than that of animals with typical granulomas (median 3.0 years, range 1.0–10.0) (p = 0.016, Mann-Whitney test).

At T2, the collected skin lesion had not healed in 22 (42%) cats, which therefore underwent a second biopsy of the same lesion. Of these 22 animals, 6 were seropositive for FeLV. Clinical cure was observed in 14 of these animals and treatment failure in 8. In the remaining 30 (58%) cats, the collected skin lesion had healed at T2 and these animals were therefore not submitted to a second biopsy. Four of these 30 cats were seropositive for FeLV and 2 were seropositive for FIV. The treatment outcome was clinical cure in 23 animals and treatment failure in 7. In this study, not only the biopsied skin lesion but all skin lesions and extracutaneous clinical signs were evaluated for the classification of treatment failure or clinical cure of the animals. There was no significant difference in the frequency of treatment outcome (p = 0.362, Fisher’s exact test) or positive serology for FIV and FeLV (p = 0.741, Fisher’s exact test) between cats undergoing one or two biopsies.

**Figure 2:**
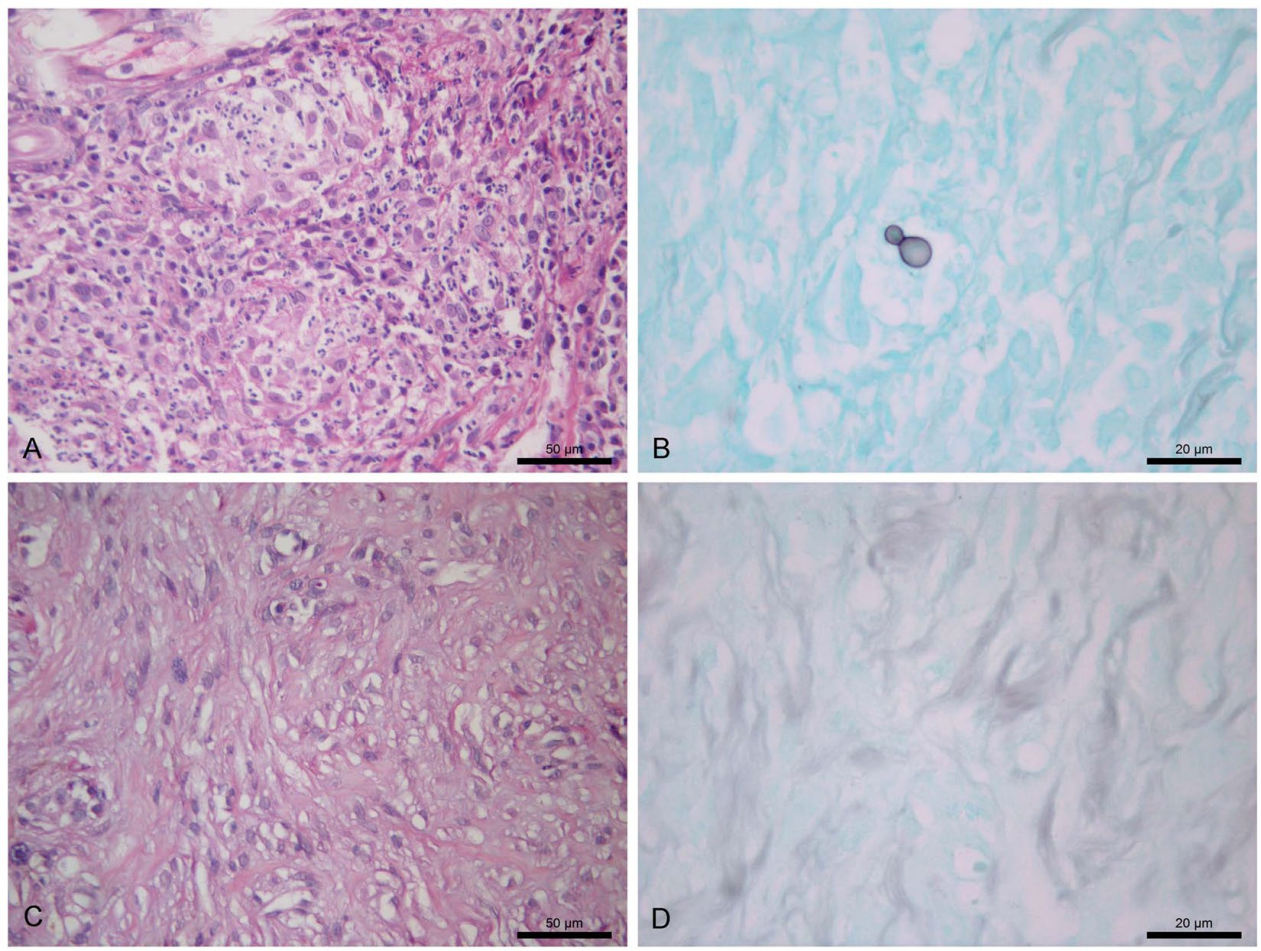
Histological changes in the skin lesion of a cat with sporotrichosis (the same as in Fig. 1A–C) whose outcome was clinical cure after 16 weeks of treatment with itraconazole. (**A**,**B**) Skin lesion sample at the time of the first biopsy before treatment. (**A**) Marked and diffuse pyogranulomatous dermatitis exhibiting multiple well-formed granulomas. H&E. (**B**) Note the black-stained single, round, budding yeast cell, demonstrating low fungal load. GMS. (**C**,**D**) Sample of the same skin lesion at the time of the second biopsy after 7 weeks of treatment. (**C**) Dermal fibrosis. H&E. (**D**) Absence of yeasts. GMS.

**Figure 3:**
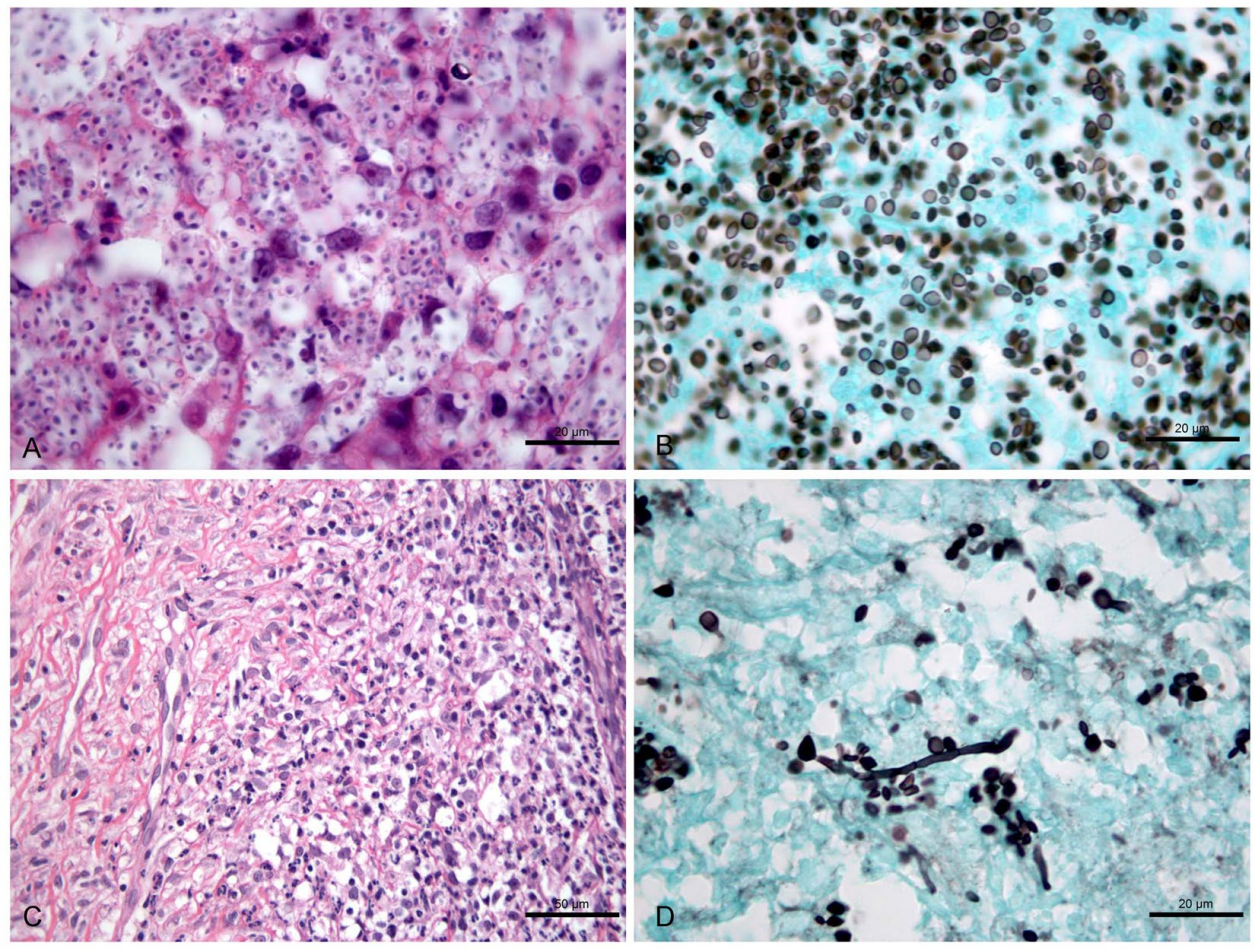
Histological changes in the skin lesion of a cat with sporotrichosis and seropositive for FIV whose outcome was therapeutic failure after 36 weeks of itraconazole treatment. (**A**,**B**) Skin lesion sample at the time of the first biopsy before treatment. (**A**) Fungus-rich granuloma containing abundant yeasts inside macrophages and few neutrophils, lymphocytes and plasma cells. H&E. (**B**) Abundant black-stained round yeasts, some with narrow-base single buds, or cigar-shaped cells. GMS. (**C**,**D**) Sample of the same skin lesion at the time of the second biopsy after 7 weeks of treatment. (**C**) Pyogranulomatous dermatitis exhibiting a poorly formed granuloma characterised by marked and diffuse infiltration of macrophages, neutrophils, plasma cells, and lymphocytes. Note also the dermal fibrosis. H&E. (**D**) Reduction in the initial fungal load showing the presence of black-stained round yeasts, some with narrow-base single buds, or cigar-shaped cells, and hyphae. GMS.

A pyogranulomatous inflammatory infiltrate was observed in the skin lesion sample biopsied at T1 of all 22 cats (100%) undergoing the second biopsy at T2. The granuloma was poorly formed in 20 (91%) cats and well-formed in 2 (9%). With respect to granuloma type, 13 (59%) animals had typical granulomas and 9 (41%) had fungus-rich granulomas. Round or cigar-shaped yeasts and hyphae or pseudohyphae compatible with *Sporothrix* spp. were detected in 21 (95%) cats. Dermal fibrosis indicating healing was not observed. Analysis of the skin lesion sample biopsied at T2 of the 22 cats showed a pyogranulomatous inflammatory infiltrate in 20 (91%) animals, with the observation of a poorly formed granuloma in 19 (95%) and of a well-formed granuloma in one (5%). Regarding granuloma type, all 20 (100%) cats exhibited typical granulomas. Two (9%) cats had a non-granulomatous inflammatory infiltrate. Round or cigar-shaped yeasts and hyphae or pseudohyphae compatible with *Sporothrix* spp. were observed in 13 (59%) animals. Dermal fibrosis indicating healing was found in 12 (54%) cats. Statistical analysis to verify the association between the histological changes observed in the skin lesion samples obtained at T1 and T2 in the group of 22 cats was not possible because all cats had pyogranulomatous inflammatory infiltrate while none had dermal fibrosis at T1. In addition, none of the cats had fungus-rich granulomas at T2. Figures 2 and 3 show the histological changes observed in skin lesion samples biopsied at T1 and T2 of a cat that was clinically cured and a cat with treatment failure, respectively. In the skin lesion sample biopsied at T2, the type of inflammatory infiltrate (p = 0.515, Fisher’s exact test), granuloma organisation (p = 1.000, Fisher’s exact test) or dermal fibrosis (p = 0.675, Fisher’s exact test) was not significantly associated with treatment outcome. Regarding granuloma type, the association with treatment outcome could not be calculated because none of the cats had fungus-rich granulomas.

Analysis of cell counts in the 22 cats undergoing the two skin biopsies showed a significant reduction in the number of macrophages and yeasts (fungal load) per mm^2^ in the skin lesion samples biopsied at T2 compared to the same lesion at T1 (Table 3). With respect to the skin lesion sample biopsied at T2, there was no difference in the number (per mm^2^) of neutrophils (p = 0.297), macrophages (p = 0.33), lymphocytes (p = 0.082), plasma cells (p = 0.525), mast cells (p = 0.402), multinucleated giant cells (p = 0.815), eosinophils (p = 0.414), or yeasts (p = 0.547) between treatment outcomes (clinical cure vs treatment failure) by the Mann-Whitney test. No difference was also found in the number of neutrophils (p = 0.381), macrophages (p = 0.677), lymphocytes (p = 0.569), plasma cells (p = 0.519), mast cells (p = 0.424), multinucleated giant cells (p = 0.677), eosinophils (p = 0.659), or yeasts (p = 0.076) between positive and negative serology for FIV and FeLV (Mann-Whitney test for all).

**Table 3:** Comparison of the number of inflammatory cells and yeasts per mm^2^ in the skin lesion sample biopsied in the first appointment before treatment with itraconazole (T1) and in the sample of the same lesion biopsied 5 to 11 weeks after the beginning of itraconazole treatment (T2) obtained from 22 cats.

Cells	Number of cells/mm^2^ in the biopsied skin lesion				p^a^
	T1 (n = 22)		T2 (n = 22)		
	Median	Range	Median	Range	
Neutrophils	148.1	47.6–239.2	132.2	0.2–341.6	0.638
Macrophages	129.0	65.4–211.4	98.0	2.4–166.2	0.036*
Lymphocytes	26.9	14–106.4	26.4	5.2–107.6	0.935
Plasma cells	11.6	0–89.2	9.3	0–140.8	0.858
Mast cells	9.4	0.6–20.8	5.0	0–27.2	0.338
MGC	0	0–3.4	0	0–2.4	1.0
Eosinophils	0	0–1.8	0	0–22.4	0.779
Yeasts	407.7	0.8–1,274.8	1.1	0–153.8	<0.001*

n, number of cats; MGC, multinucleated giant cells; ^a^Wilcoxon signed-rank test. *Significant difference (p < 0.05).

## Discussion

The emergence of *S. brasiliensis* in Brazil brought new challenges to the control of sporotrichosis and determined the domestic cat as the most susceptible host and the main source of infection in an area of unprecedented hyperendemic occurrence of this zoonosis. In this context, this study investigates for the first time, before and during the treatment, clinical and histological predictors for the itraconazole treatment response in cats with sporotrichosis caused by *S. brasiliensis* and provides new information for the improvement of the treatment of cats and control of this zoonosis.

The identification of *S. brasiliensis* in all cats of the present study corroborates the findings of other authors^10,11^ reported for cats from the same region. Although this fungal species is considered the most virulent within the genus^19,20^, the response to treatment with itraconazole was satisfactory in the animals studied, with clinical cure of more than two-thirds of the animals after a median treatment time of 16 weeks. Furthermore, a significant reduction in the number and distribution of skin lesions, fungal load and number of macrophages in the lesions examined was observed within 5 to 11 weeks after the beginning of treatment. The presence of melanin in conidia (pigmented conidia), which is an important virulence factor^21^, did not influence the response to treatment with itraconazole in this study.

The cats included in this study were from the same geographic region and had similar clinical characteristics as the cats of a previous similar study^16^. However, in that study^16^, only 38% of the animals treated with similar doses of itraconazole were clinically cured after a median treatment time of 26 weeks. These values show a much lower treatment response than that found in this study and may be explained by the different study designs and inclusion of cats whose owners did not adhere to the treatment protocol and cats that died during treatment, but whose cause of death was not confirmed as sporotrichosis^16^.

Respiratory signs and nasal mucosa lesions were detected in the same cats at the first appointment and at 5 to 11 weeks of treatment and were later found to be associated with failure of itraconazole treatment, in agreement with the results of another study^16^. The severity of the inflammatory reaction, high fungal loads and occurrence of lesions in the nasal mucosa, cartilage and bone of cats infected with *Sporothrix* spp.^3^, as well as the lack of a rich blood supply in the nasal planum^22^, may explain the difficult healing of lesions at this anatomical site. Nevertheless, in the present study, regular treatment with itraconazole at the dose used was found to promote clinical cure in slightly more than half (59%) of the animals with respiratory signs and nasal mucosa lesions. One alternative to increase the rates of clinical cure in cats with sporotrichosis that exhibit respiratory signs and nasal mucosa lesions is the combination of itraconazole with potassium iodide^23,24^.

In the present study, the frequency of cats with positive serology for FIV or FeLV was similar to the 25% reported by other authors^11,25^ and slightly higher than the frequencies described in other studies, 19%^26^ and 22%^15^, for cats with sporotrichosis from the same region. As reported in those studies, we also found no association between infection with these retroviruses and treatment outcome. However, the histological changes observed in skin lesion samples of coinfected cats suggest a less efficient immune response when compared to non-coinfected cats, with a consequently lower capacity to eliminate the fungus. These histological changes were a significantly smaller number of neutrophils, as well as a smaller number of macrophages, higher fungal load and absence of well-formed granulomas, although not statistically significant when compared to non-coinfected cats. One explanation for these findings is that FIV and FeLV are immunosuppressive viruses that can cause neutropenia alone or in conjunction with other cytopenias^27,28^. FeLV can also reduce the phagocytic and chemotactic function of neutrophils and can cause deficient healing of skin lesions^28,29^. Neutrophils and activated macrophages, which produce superoxide anion and its reactive oxygen metabolites, play an important role in phagocytosis and in the elimination of *Sporothrix* spp.^30–32^. The fungicidal activity of these inflammatory cells is fundamental for the success of itraconazole, which is primarily fungistatic^33^. Additionally, the fungicidal activity of these inflammatory cells can explain the inverse relationship between the number of neutrophils and macrophages and fungal load observed in the present study in lesions before the beginning of treatment. Other authors^17^ also reported an inverse relationship between neutrophils and fungal load in feline sporotrichosis. Neutrophils are also important for wound healing by removing dead cells and bacteria through phagocytosis, as well as by releasing angiogenic growth factors, expressing proteases that are important for tissue repair, and facilitating the recruitment of monocytes to the site of inflammation^34^. This participation of neutrophils in the healing of lesions was demonstrated in the present study by the inverse relationship between neutrophils and healing time. However, the lack of serological monitoring of infection with FIV and FeLV and of the immune response during treatment, as well as the small number of seropositive cats, were limitations of this study. These limitations may have impaired confirmation by statistical analysis of the influence of positive serology for FIV and FeLV on treatment outcome and wound healing time in feline sporotrichosis.

The predominance of poorly formed granulomas and fungus-rich granulomas was similar to the histological changes in the skin reported by other authors^3,17^ for untreated cats with sporotrichosis from the same region. These results suggest that the immune response to *S. brasiliensis* was not efficient in eliminating the infection in most of the cats examined. This hypothesis is supported by the fact that the fungal load was significantly higher in cats with skin lesions containing poorly formed granulomas and fungus-rich granulomas. Additionally, a high fungal load (median of 745 yeasts/mm^2^) was associated with treatment failure and correlated with a longer healing time of the lesion examined. On the other hand, the results of this and of another study^17^ indicated a better control of fungal load for well-formed granulomas. The latter granulomas are related to a cell-mediated immune response (Th1) and are more frequent in dogs and humans, which generally develop less severe forms of sporotrichosis and show a better response to antifungal treatment than cats^2,17,18,35,36^. Although all cats of this study with well-formed granulomas were cured, no significant association was found between this histological change and treatment outcome. This result is probably due to the small number of cats with well-formed granulomas before the beginning of treatment.

The present results showed that young cats tend to have fungus-rich granulomas, while adult animals tend to have typical granulomas. Additionally, a higher fungal load and smaller number of lymphocytes were correlated with younger age of the animals. The number of lymphocytes, macrophages and neutrophils was directly correlated with a reduction in fungal load in the present study. In agreement with these results, other authors^17^ studying feline sporotrichosis observed a significantly higher intensity of lymphocyte infiltration and a higher frequency of well-formed granulomas in cats older than 5 years compared to younger animals. Taken together, these findings suggest that the immune response against the fungus *Sporothrix* spp. is less efficient in young cats.

The results of this study indicate that itraconazole is effective for the treatment of feline sporotrichosis caused by *S. brasiliensis*. However, the occurrence of respiratory signs, nasal mucosa lesions and skin lesions with high fungal loads and fungus-rich granulomas are predictors of treatment failure. A high fungal load in skin lesions of cats was linked to younger age and histological skin changes suggestive of an immunodeficient response, such as poorly formed granulomas and a reduced number of macrophages, neutrophils and lymphocytes. The causes of these alterations should be investigated. Additionally, a smaller number of neutrophils and a higher fungal load may impair the healing of skin lesions in cats with sporotrichosis. Altogether, these findings highlight the importance of further investigation of appropriate strategies for conducting the treatment of feline sporotrichosis, particularly in cats that present the predictors for treatment failure herein defined.

## Methods

### Sample

The population of this study consisted of a non-probability sample of 52 cats treated at the Evandro Chagas National Institute of Infectious Diseases, Oswaldo Cruz Foundation, Rio de Janeiro, Brazil, between August 2011 and December 2013. Cats of both sexes weighing more than 2 kg and aged more than 12 months and less than 12 years were included in the study. Additional inclusion criteria were the presence of skin lesions, a definitive diagnosis of sporotrichosis by isolation of *Sporothrix* spp. in culture media, and the absence of previous treatment with antifungal drugs and pregnancy. Owners who agreed to participate in the study signed an informed client consent form.

### Clinical examination and sample collection

In the first appointment (T1), the cats were submitted to complete clinical examination and collection of a blood sample and representative samples of the skin lesion(s). The presence of skin and nasal mucosa lesions, presence of respiratory signs (sneezing, nasal discharge, or dyspnoea), and body condition were evaluated during clinical examination. The animal’s body condition was classified as good, fair, or poor^17^. The skin lesions were measured, counted and classified according to their distribution: L1 (lesions at only one site), L2 (lesions at two non-contiguous sites), and L3 (lesions at three or more non-contiguous sites)^15^.

The blood and skin samples were collected after sedation of the animals by intramuscular injection of 1% acepromazine (0.1 mg/kg) and 10% ketamine hydrochloride (10–15 mg/kg). Peripheral blood samples were collected by venipuncture and serum samples were obtained for the diagnosis of FIV and FeLV. For collection of the skin lesion samples, one site was selected using the criterion of greater extent in the case of multiple lesions; however, preference was given to lesions on the nasal planum because they are reportedly more difficult to heal^3^ and are therefore more likely to allow a subsequent biopsy. First, an exudate of the selected active skin lesion was collected with a sterile swab for mycological culture. For cytopathological examination, impression smears were prepared on clean and dry glass slides for preliminary diagnosis so that antifungal therapy could be initiated. Two 3–4 mm punch biopsies were obtained from the border of the selected active skin lesion after local asepsis with 70% alcohol and anaesthesia with 2% lidocaine. One biopsy sample was fixed for 48 h in 10% neutral buffered formalin for histopathological examination. The other biopsy sample was kept in sterile saline solution containing antibiotics for mycological culture.

### Cytopathological examination

The impression smears were stained by the Quick Panoptic method and analysed by light microscopy^37^.

### Mycological culture

The exudate and biopsy skin sample were seeded in Petri dishes containing Mycosel agar. The plates were incubated at 25 °C and examined for 30 days. The isolates suspected of being *Sporothrix* spp. were subcultured on potato dextrose agar (PDA) at 25 °C for morphological identification. The dimorphism of the fungus was verified by the growth of yeast-like forms in brain heart infusion (BHI) medium at 37 °C^15^.

### Identification of *Sporothrix* species

The fungal cultures in the filamentous phase were seeded in Petri dishes containing PDA and incubated at 30 °C and 37 °C for 21 days in the dark for the observation of macroscopic characteristics of the colonies. Microscopic morphology and conidial pigmentation were analysed by culture on microscope slides^38^ in corn meal agar for 12 days at 30 °C in the dark. The assimilation of glucose, sucrose, raffinose and ribitol was tested using a previously described method^4^.

Isolates that were not identified to species level by phenotyping as described above were submitted to DNA extraction^9^ for PCR fingerprinting with the T3B primer^39^.

### Serological diagnosis of FIV and FeLV coinfection

Serum samples obtained from 48 of the 52 cats included in the study were tested for the presence of antibodies to FIV and FeLV antigens by enzyme immunoassay using the SNAP Combo FeLV/FIV test (IDEXX Laboratories) according to manufacturer instructions.

### Antifungal therapy

The treatment of the cats was started at T1 when cytopathological examination was positive for yeast-like forms suggestive of *Sporothrix* spp. In the case of cats with negative cytopathological examination at T1, treatment was initiated when mycological examination was positive for *Sporothrix*, which occurred 5 to 30 days after T1.

The animals were treated with 50 or 100 mg oral itraconazole (capsules) every 24 h. The drug was administered with food by the owners at home. In the case of complete remission of all skin lesions and extracutaneous signs of sporotrichosis (i.e., dyspnoea, sneezing, nasal discharge, and regional lymphadenomegaly) at the monthly recheck, the animal was treated for an additional 8 weeks. The maximum treatment duration was set at 36 weeks, which is the reported median time of treatment to achieve cure in cats from the same region^15^.

Criteria for treatment discontinuation or withdrawal of the animal from the study were loss to follow up characterised by non-attendance of the regular rechecks on two consecutive appointments, decision of the owner, and death due to causes unrelated to sporotrichosis.

### Follow-up

The clinical response to therapy was monitored by monthly rechecks from the beginning to the end of itraconazole therapy. One additional biopsy of the same skin lesion as the first one was taken between the 5th and 11th week after the beginning of treatment (T2) to determine progression of the histological changes in the skin. If the skin lesion had healed at T2, the second biopsy was not taken. The time of the second biopsy was chosen based on the reported minimum period of 8 weeks^15^ to achieve clinical cure with itraconazole and included a variation of 3 weeks due to difficulties in scheduling the rechecks. The skin sample with the lesion biopsied at T2 was processed for histopathological examination and mycological culture as described for the first biopsy. At T2, the cats were also submitted to complete clinical examination as described above for T1, except for the measurement of skin lesions. No peripheral blood sample was collected at T2.

### Classification of clinical outcomes

The clinical outcomes were classified as clinical cure (cats with complete remission of skin lesions and extracutaneous signs of sporotrichosis during the follow-up period) or treatment failure (cats that showed stagnation or worsening of skin lesions and extracutaneous signs in two consecutive monthly rechecks, and cats not clinically cured after 36 weeks of treatment with itraconazole).

### Evaluation of histological changes and fungal load

The skin biopsy samples fixed in 10% neutral buffered formalin were processed for routine paraffin embedding, sectioned at 5 µm, and stained with haematoxylin and eosin (H&E), Grocott’s methenamine silver stain (GMS) and Giemsa^40^.

The skin inflammatory infiltrate was classified as pyogranulomatous in the case of a predominance of macrophages and large number of neutrophils, and as non-granulomatous when other cell types predominated^17^. The organisation of the granulomatous infiltrate was classified as well- or poorly formed^17^ and the type of granulomas was classified as fungus-rich granuloma or typical granuloma. The fungus-rich granuloma (synonym of fungal granuloma)^17^ was a poorly formed granuloma containing macrophages packed with yeasts and a sparse lymphoplasmacytic infiltrate^15^. Both the poorly formed granuloma without the characteristics of fungus-rich granulomas described above and the well-formed granuloma were classified as typical granuloma. The cell types in the inflammatory infiltrate were recorded and quantified with the aid of a 1-mm^2^ optical grid reticle and a manual cell counter. The cells were counted in H&E- and Giemsa-stained (only mast cells) sections in five high power grid fields (400x) in the most cellular areas of the lesion and the number of cells number was averaged. Intralesional yeasts were quantified in GMS-stained sections using the same method.

### Statistical analysis

Statistical analysis was performed using the Statistical Package for the Social Sciences for Windows (version 16.0). Qualitative variables are reported as frequencies and quantitative variables as median and range.

Fisher’s exact test was used to evaluate the association of qualitative variables according to treatment outcome and positive serology for FIV/FeLV. The quantitative variables were not normally distributed (Shapiro-Wilk test, p < 0.05) and differences in these variables according to treatment outcome and positive serology for FIV/FeLV were therefore analysed by the nonparametric Mann-Whitney test. Spearman’s rank correlation coefficient was used to evaluate the correlation between quantitative variables.

The histopathological results (number of cells) of skin samples from the same lesion biopsied at T1 and T2 were compared by the nonparametric paired Wilcoxon signed-rank test. The paired McNemar test was used to compare the qualitative variables between T1 and T2 (body condition, distribution of skin lesions, presence of respiratory signs, and nasal mucosa lesions). A p value < 0.05 indicated statistically significant results.

### Ethics statement

The study protocol was approved by the Ethics Committee on Animal Use of the Oswaldo Cruz Foundation (CEUA/FIOCRUZ; Permit Number: LW-25/11). This study was carried out in strict accordance with Brazilian law number 11.794 of 2008, which regulates the care and use of animals in teaching and research in Brazil.

### Data availability

All data generated or analysed during the current study are included in this article.
